# Supporting Weight Loss Among Parents of Children With a Disability: Lessons Learned From a Single-Arm Pilot Study

**DOI:** 10.2196/63858

**Published:** 2024-10-07

**Authors:** Payson Wisniewski, Julia Depuy, Cassandra Kim, Olivia Garrison, Gerald J Jerome

**Affiliations:** 1 Department of Kinesiology Towson University Towson, MD United States

**Keywords:** weight loss, obesity, disability, parent, family, child, weight loss intervention

## Abstract

This study assessed weight change in the parents of children with disabilities following a 12-week, remotely delivered weight loss program focused on lifestyle modifications and found a significant median weight reduction of 3 kg from baseline to week 12.

## Introduction

Engaging parents is important when addressing childhood obesity [1]. In fact, a parent-only approach, where the weight loss program focuses on helping the parent lose weight and monitors weight change in the child holds potential to benefit both the parent and the child [2,3]. In this approach, the parent models healthy behaviors and makes decisions for the household (eg, shopping, meal preparation, active family outings) that would also support the child’s weight management. This approach holds particular benefit for families that include a child with a disability. In these families, not only are the children at risk of being excluded from more mainstream weight loss solutions, but parents with children who have a disability or health problem have reported high levels of mental and physical health problems and a need for self-care assistance [4-6]. Developing a weight loss program for the parents of children with a disability is an important next step in providing assistance to these families. This single-arm pilot study examined weight change from a 12-week, remotely delivered weight loss program among parents who have a child with a disability.

## Methods

### Overview

Participants were recruited through postings with advocacy groups. The study was conducted remotely using email, a web-based Qualtrics survey, and video conference sessions with study staff. Eligibility requirements included being a parent (aged ≥18 years), with a BMI ≥25 kg/m², who had a child (aged 8-18 years) with a mental or physical disability that was supported with an Individualized Education Plan or 504 Plan at school. We excluded individuals with a diagnosed eating disorder, current or planned pregnancy, or planned weight loss surgery. The primary aim was to examine the 12-week weight change of the parent. We also examined program adherence.

 Parents self-reported demographic information (February 2021 to December 2022) and scales that used cellular technology sent weight data to the research team for both data collection (at baseline, weeks 12 and 24) and accountability during the weight loss program.The 12-week weight loss program included weight loss materials sent via email, weekly video-based coaching calls, and weekly tailored emails. Participants received evidence-based lifestyle recommendations of regular self-weighing, decreasing caloric intake, gradually increasing physical activity to 150 minutes/week, tracking of these behaviors, and an initial weight loss goal of 5 pounds. Coaches worked with the participants to identify food or drink that was calorically dense, of low nutritional value (eg, sugar beverages, fast foods, baked goods), and eaten frequently (eg, daily). Then coaches worked with the participants to identify strategies to eliminate those items from their diet. Similar weight loss programs were successful in previous pilot studies [7,8].

### Ethical Considerations

All parents provided informed consent, the study was in accordance with the Declaration of Helsinki, and the protocol was approved by the Towson University Institutional Review Board (1409, 1181).

## Results

We screened 33 parents and enrolled 13. Participants were female, with a median age of 44 (IQR 42.5 to 48.5) years, and 76.9% (10/13) were non-Hispanic White (Table 1). Three participants discontinued the weight loss program (in weeks 3, 8, and 12). Table 2 shows compliance with weekly coaching calls (median 12, IQR 10.5 to 12) and weight tracking (median 12, IQR 10 to 12). Completer analyses using the Wilcoxon signed-ranked test (α=.05) showed a significant reduction in weight from baseline to week 12 (end of the program; n=10, median –3, IQR –5.2 to –1.4 kg; *P*=.02) but not week 24 (follow-up; n=10, median –3.9, IQR –4.9 to –0.7 kg; *P*=.07).

**Table 1:** Participant characteristics.

		Parent (N=13)	Child (N=13)
**Age (years), median (IQR)**		44 (42.5-48.5)	12 (11-16.5)
**Weight (kg), median (IQR)**		88.8 (80.9-105.6)	—^a^
**BMI (kg/m^2^), median (IQR)**		34 (29.7-39.9)	—
**Sex, n (%)**			
	Female	13 (100)	10 (76.9)
	Male	0 (0)	3 (23.1)
**Race and ethnicity, n (%)**			
	Non-Hispanic White	10 (76.9)	8 (61.5)
	Non-Hispanic Black	2 (15.4)	2 (15.4)
	Hispanic	0 (0)	2 (15.4)
	Other	1 (7.7)	1 (7.7)

^a^Not applicable.

**Table 2:** Participant compliance and program effectiveness.

		End of program (week 12)		Follow-up (week 24)
**Weekly coaching calls completed, median (IQR)**				
	All participants (N=13)		12 (10.5 to 12)	—^a^
	Completers (n=10)		12 (12 to 12)	—
**Weeks with at least 1 weight tracked, median (IQR)**				
	All participants (N=13)		12 (10 to 12)	—
	Completers (n=10)		12 (12 to 12)	—
**Weeks with some diet tracking, median (IQR)**				
	All participants (N=13)		12 (10 to 12)	—
	Completers (n=10)		12 (11.8 to 12)	—
**Weeks with some physical activity tracking, median (IQR)**				
	All participants (N=13)		12 (11 to 12)	—
	Completers (n=10)		12 (12 to 12)	—
**Weight change (kg), median (IQR)**				
	Completers (n=10)		–3 (–5.2 to –1.4)	–3.9 (–4.9 to –0.7)
**Weight change (%), median (IQR)**				
	Completers (n=10)		–3.6 (–5.5 to –1.6)	–4.1 (–5.6 to –1.1)
**Achieved 5 lbs weight loss goal, n (%)**				
	Completers (n=10)		7 (70)	6 (60)

^a^Not applicable.

## Discussion

This was a single-arm pilot study of a 12-week weight loss program for parents with a child who has a disability. Statistically significant short-term weight loss demonstrated the feasibility of this remotely delivered program. There was high compliance with coaching calls and weight tracking, suggesting the parents were interested in weight loss. However, program improvements are needed to reduce dropouts and enhance long-term weight loss for all participants. Both completers with weight gain (data not shown) were single parents. The burden of childcare varies greatly across disabilities [5] and can limit the time available for parental self-care, especially for single parents. Solutions tailored to family needs may include providing healthy meals or childcare to allow parents time to engage in recommended lifestyle changes. In addition, mothers of children with disabilities have expressed the need for mental health support but find it challenging to get assistance [9]. Programming that supports the mental and physical health of the parents by combining stress management with weight loss management could lead to improved weight loss [10]. This was a small study without a control group, yet the results support the further development of a weight loss program to help these parents with long-term weight management.

## Data Availability

Participants granted the study team permission to report aggregated data. None of the participants granted permission for data sharing.
